# Microbeam Radiation Therapy Bio-Dosimetry Enhanced by Novel Radiosensitiser Combinations in the Treatment of Brain Cancer

**DOI:** 10.3390/cancers16244231

**Published:** 2024-12-19

**Authors:** Michael Valceski, Elette Engels, Sarah Vogel, Jason Paino, Dylan Potter, Carolyn Hollis, Abass Khochaiche, Micah Barnes, Alice O’Keefe, Matthew Cameron, Kiarn Roughley, Anatoly Rosenfeld, Michael Lerch, Stéphanie Corde, Moeava Tehei

**Affiliations:** 1 Centre for Medical Radiation Physics, University of Wollongong, Wollongong, NSW 2522, Australia; 2 Molecular Horizons, University of Wollongong, Wollongong, NSW 2522, Australia; 3 Australian Synchrotron-Australia’s Nuclear Science and Technology Organisation (ANSTO), 800 Blackburn Road, Clayton, Melbourne, VIC 3168, Australia; 4 Department of Physical Sciences, Peter MacCallum Cancer Centre, Melbourne, VIC 3000, Australia; 5 Radiation Oncology Department, Prince of Wales Hospital, Randwick, Sydney, NSW 2031, Australia

**Keywords:** synchrotron, microbeam radiation therapy, nanoparticles, chemotherapy, radiotherapy, DNA damage, bio-dosimetry, peak-to-valley dose ratio, γH2AX, confocal microscopy

## Abstract

**Background/Objectives**: Brain cancer is notoriously resistant to traditional treatments, including radiotherapy. Microbeam radiation therapy (MRT), arrays of ultra-fast synchrotron X-ray beams tens of micrometres wide (called peaks) and spaced hundreds of micrometres apart (valleys), is an effective alternative to conventional treatments. MRT’s advantage is that normal tissues can be spared from harm whilst maintaining tumour control. Combining MRT with targeted radiosensitisers, such as nanoparticles, chemotherapeutic drugs, and halogenated pyrimidine drugs, can further improve radiotherapy by enhancing radiation damage. However, the underlying mechanisms of MRT are still being understood, which is essential to ensuring the reliable and successful use of MRT. **Methods**: An in vitro study was performed using γH2AX imaging, and quantification was performed via confocal microscopy and a clonogenic cell survival assay. **Results**: We show that methotrexate chemotherapeutics and iododeoxyuridine enhance MRT cell-killing and thulium oxide nanoparticles (TmNPs) broaden MRT peaks, and using γH2AX immunofluorescent confocal microscopy to quantify DNA damage, we further our knowledge of MRT mechanisms. γH2AX images verify the biological responses of cells aligning with the physical collimation of MRT, and we can accurately measure MRT microbeam characteristics bio-dosimetrically. The peak-to-valley dose ratio (PVDR), the ratio of the peak dose to the valley dose that characterises an MRT field, was accurately measured biologically using γH2AX imaging, despite studies previously finding this challenging. **Conclusions**: The measurement of biological PVDR has been performed for the first time with high-Z radiosensitisers, including nanoparticles, and several novel radiosensitiser-enhanced MRT mechanisms were discovered. Our results deepen our understanding of MRT with radiosensitisers, and can contribute to its accurate and future successful use in treating cancer.

## Simple Summary

Brain cancer is resistant to conventional treatments, including radiotherapy. Microbeam radiation therapy (MRT), arrays of synchrotron X-ray beams tens of micrometres wide (called peaks) and spaced hundreds of micrometres apart (valleys), provides an alternative modality, advantaged by normal tissue-sparing whilst maintaining tumour control. Combining MRT with radiosensitising nanoparticles, chemotherapeutic drugs, and halogenated pyrimidine drugs can further improve radiation damage. However, the mechanisms of MRT are still being understood. Here, we show that methotrexate chemotherapeutics and iododeoxyuridine enhance cell-killing and thulium oxide nanoparticles broaden peaks, and using γH2AX confocal microscopy, we better understand MRT’s mechanisms. γH2AX images verify the biological responses of cells aligning with physical MRT collimations, and we can accurately measure MRT microbeam characteristics bio-dosimetrically. The peak-to-valley dose ratio, which characterises an MRT field, was accurately measured biologically using γH2AX imaging. Our results deepen our understanding of MRT with radiosensitisers and can contribute to its successful use in treating cancer.

## 1. Introduction

Cancer is a family of diseases resulting in millions of deaths each year [1], and glioblastoma multiforme (GBM), which is a primary brain glioma, has both the highest incidence and the lowest survival rates [2,3]. 9L gliosarcoma (9LGS), a GBM variant, is a rodent cell model for these brain cancers that is commonly used for in vitro studies of GBM [1,4,5,6]. Like most GBM cancers, 9LGS cells are similar to high-grade human glioma cells and are highly resistant to conventional cancer treatment [4,7].

Surgery, chemotherapy, and radiotherapy (RT) remain the most common methods for cancer treatment, yet each faces limitations. This includes difficulty in safe surgical resection, whilst drugs can also affect normal tissues [8,9,10,11,12,13]. Radiation treatments use ionising radiation (IR) to target and damage tumours directly or indirectly through the production of secondary radiations (e.g., ionized electrons) that induce DNA damage [7,8,10,11].

The two main types of DNA damage following irradiation are single-strand DNA breaks (SSB), in which only one strand of the DNA double helix is broken (comprising the majority of breaks), and double-strand DNA breaks (DSB), in which both strands are severed (which is the most lethal) [14,15,16,17]. RT also faces the challenge of controlling tumours whilst minimising exposure to nearby healthy tissues, which can also experience DNA damage from RT [7,10]. Radiation doses are then restricted by normal tissue tolerance, so improved methods of targeted RT are needed.

One method is the ultra-fast delivery of therapeutic radiation doses at rates many times greater than conventional RT methods that have been observed in living cells, with radiotherapeutic doses delivered at rates ≥ 40 Gy/s [18,19,20,21]. These ultra-high dose rate (UHDR) effects have the benefit of protecting healthy tissues whilst maintaining tumour control, notably by reducing toxicity to normal cells exposed to IR during the RT regimen [18,19,20,21]. This is also seen with brilliant, high-flux, UHDR synchrotron radiation and spatially fractionated microbeams [18,19,20,21,22].

Produced by synchrotron particle accelerators with strong magnetic fields, synchrotron broadbeam (SBB) collimations can be reconfigured into another novel modality. Synchrotron microbeam radiation therapy (MRT) involves the use of spatially fractionated arrays of UHDR X-rays that can be collimated into beams tens of microns wide and spaced hundreds of microns apart [18,21,22,23,24]. The regions of MRT fields experiencing the full microbeams are described as peaks, whilst the regions between are defined as valleys, allowing this array to be characterised with a ratio of doses delivered to peaks over valleys, called the peak-to-valley dose ratio (PVDR) [18,25].

Whilst physical PVDR is accurately determined in many MRT studies [26,27], many studies have also attempted, somewhat unsuccessfully, to link the biological effects to the physical MRT prescribed. This has included attempts to obtain a biological PVDR measurement, including through the use of DNA damage assays, such as γH2AX, that match the physical value [28,29]. However, some studies concluded that it was not possible to reliability determine a biological PVDR equivalent, or faced limitations or observed variations in their acquired values due to various potential factors [28,29]. However, our previous work developing an alternative γH2AX analysis method provided a means of verifying PVDR biologically, which can be used in future MRT studies to further improve the benefits of MRT [30].

Slatkin et al. describe the advantage of MRT being tissue-sparing of normal tissues, including following the irradiation of Fischer 344 rat brains that displayed no evidence of MRT-induced necrosis or brain damage [22,23,24]. Synchrotron MRT has been observed to control cancer both in vitro and in vivo at kilovoltage energies and high dose rates [18,22,23,24]. Engels et al. show the effect of both SBB and MRT, whereby HDR effects were found to significantly reduce 9LGS cell survival and improve Fischer 344 rat survival following the image-guided MRT treatment of 9LGS tumours [22].

Some studies have attempted alternative methods of improved cancer targeting and RT, including nanotherapy, whereby nanoparticles (NPs) measuring in typical size from 1 to 100 nm have become popular due to their easy passage into cells [12,31,32,33]. NPs can enhance radiation dose and cancer damage in RT, in which high-Z materials are favoured due to their abundant atomic electrons available for photon interactions. NP enhancement is greatest with kilovoltage (kV) energies, in which photoelectric interactions dominate and many secondary electrons are produced, resulting in lethal genetic lesions [34,35].

Many NPs have been investigated for their therapeutic capabilities, such as gold NPs and tantalum oxide and gadolinium oxide NPs, including in 9LGS tumours in vitro and in vivo, with promising results [12,34,36,37,38,39]. Thulium oxide (Tm_2_O_3_, Z = 69) NPs (TmNPs) are a useful candidate previously characterised for kV peak (kVp) energies with a conventional broadbeam (CBB) orthovoltage device by Engels et al. [7]. TmNPs were observed to reduce cancer cell survival and increase DSB incidence in 9LGS cells, making these cytoplasm-localised NPs an appropriate candidate for continued investigation as a radiosensitiser [7], including with HDR MRT.

Another option is combining chemotherapeutic drugs with RT. Halogenated pyrimidine drugs (nucleoside analogues bound to halogen elements) are useful anti-cancer drugs due to their disruption and replacement of DNA segments [40,41]. High-Z halogens can deliver additive effects combined with RT, such as iodine (I, Z = 53) forming iododeoxyuridine (IUdR). DNA-localised radiosensitisers have the potential for substantial dose enhancement in cancer cells via photoelectric effect due to their proximity to DNA, in which low energy secondary electrons can produce localised DNA damage [39,42,43,44]. The exposure of even 10 µM of these types of drugs is used with great effect on cells exposed to kVp CBB radiation [39,44,45]. Corde et al. observed a significant enhancement using SBB X-rays at similar energies with cells exposed to IUdR prior to irradiation [42], making IUdR a desirable candidate for continued investigation and comparison with TmNPs.

A purely chemotherapeutic option is methotrexate (MTX), a well-established anti-cancer drug that inhibits the production of folate, which in turn disrupts cellular proliferation and DNA synthesis and repair [44,46]. Folate surface receptors are generally over-expressed on cancer cells, including brain cancer, allowing for a potential affinity for tumour cells over normal tissues [47,48]. These characteristics make MTX another useful candidate for cancer radiosensitisation, given that the cellular response to radiation damage could be greatly inhibited [46].

MTX could then be combined with high-Z radiosensitisers, TmNPs, and IUdR for additive effects to synergistically amplify radiation dose and cancer-killing even further. Given the tissue-sparing effects of novel HDR RT methods, including MRT, this study will explore an opportunity to combine multiple treatment modalities for a synergistic effect and use it to bio-dosimetrically measure the biological response to this multi-modal radiotherapy. Accordingly, this study will focus on the synergistic potential of MRT using IUdR+MTX and Tm+MTX and will not only investigate the efficacy of MRT in treating GBM brain cancer, such as 9LGS, but also explore the underlying mechanisms. This investigation will provide a deeper look into the mechanisms behind the MRT treatment of cancer, including a radiobiological analysis that can be matched to the physical treatment delivered. Particularly, as previous studies have investigated [28,29], we will also look at improving our ability to effectively use MRT, including with radiosensitisers, by determining biological PVDR values for all treatment combinations. We then hypothesize that the use of radiosensitisers, such as IUdR+MTX and Tm+MTX, will result in improved cancer-killing due to local dose enhancement in 9LGS cells, which would in turn reduce the biological PVDR. Consequently, such enhancement would permit a lower MRT dose to be used in a fraction to achieve the same efficacy, thereby improving the tissue-sparing capabilities of MRT whilst maintaining tumour control.

## 2. Materials and Methods

### 2.1. Subculture of Adherent Cells

9L gliosarcoma (9LGS) cells, obtained from the European Collection of Cell Cultures (ECACC), were cultured in Greiner Bio-One T75 cm^2^ flasks (Interpath, Melbourne, VIC, Australia, #658175) containing complete Gibco Dulbecco’s Modified Eagle Medium (c-DMEM) (DMEM; Brisbane, QLD, Australia, #11965118), with added Gibco 10% foetal bovine serum (FBS; ThermoFisher Scientific, Brisbane, QLD, Australia, #10099141), and Gibco 1% penicillin (10,000 units/mL), and streptomycin (10,000 μg/mL) (ThermoFisher Scientific, Brisbane, QLD, Australia, #15140122). Cultures were incubated at 37 °C and 5% (*v*/*v*) CO_2_. The 9LGS cell doubling time was 36 h.

When passaged or harvested, cells are washed with Gibco 1x Dulbecco’s Phosphate Buffered Saline (DBPS; Ca^2+^/Mg^2+^ free; ThermoFisher Scientific, Brisbane, QLD, Australia, #14190144) before being suspended with Gibco 0.05% trypsin EDTA (ThermoFisher Scientific, Brisbane, QLD, Australia, #25300054). 9LGS cells were harvested via this passaging method and counted and seeded as monolayers into T12.5 cm^2^ flasks (Corning Incorporated, Corning, NY, USA, #353107) or Ibidi 1 cm^2^ (well area) micro-chamber slide wells (DKSH Australia, Sydney, NSW, Australia, #80827). Cells were treated when they reached 100% confluence.

### 2.2. Nanoparticle Preparation

Thulium (III) oxide (Tm_2_O_3_) nanoparticles (99.9% trace metals basis) were obtained from Sigma Aldrich (via Merck Life Science, Melbourne, VIC, Australia, #289167). The Tm_2_O_3_ NPs (TmNPs) were sonicated for 40 min in DPBS (Ca^2+^/Mg^2+^ free, Gibco, Australia) at a concentration of 1 mg/mL (*w*/*v*) to separate particles using an ultrasonic water bath (Branson). Following the protocol of Engels et al. [7], the NPs were then added to samples at a concentration of 50 μg/mL 24 h prior to the cells reaching 100% confluence.

As the TmNPs were observed to sink within the cell culture flask, the 50 µg/mL volumetric concentration corresponded to a 20 µg/cm^2^ area density in the T12.5 cm^2^ flask, which was used for cell survival studies in response to radiation treatment. As such, this area concentration was then used in micro-slide wells for consistency and correlation with the survival data.

### 2.3. Halogenated Pyrimidine Preparation

Iododeoxyuridine (IUdR) powder stock was obtained from Sigma-Aldrich (≥99% HPLC; Merck Life Science, Melbourne, VIC, Australia, #I7125) and diluted in Gibco Hank’s Balanced Salt Solution (HBSS; no phenyl red; ThermoFisher Scientific, Brisbane, QLD, Australia, # 14175103) for a stock solution of 1.6 mg. Initial and intermediate dilutions for lower concentrations were produced in c-DMEM. These dilutions were added to samples to produce a final concentration of 10 μM IUdR with two doubling times prior to cells reaching 100% confluence.

### 2.4. Chemotherapeutic Drug Preparation

Methotrexate (MTX) powder stock was obtained from Sigma-Aldrich (Merck, Melbourne, VIC, Australia, #M8407). A dilution of 2 mg/mL of MTX was prepared with 2 M of NaOH (Sigma-Aldrich via Merck Life Science, Melbourne, VIC, Australia, #S5881) in Gibco HBSS buffer (with phenyl red; ThermoFisher Scientific, Brisbane, QLD, Australia, #24020117), for a stock solution with a pH of 9.5. Intermediate dilutions at lower concentrations were produced in HBSS. These dilutions were added to samples to produce a final concentration of 0.01 μM MTX with two doubling times prior to 9LGS cells reaching 100% confluence.

### 2.5. Addition of Radiosensitiser Combinations

When TmNPs are combined with MTX (Tm+MTX), the MTX is added the day after flask or slide seeding when the cells have settled and adhered (two doubling times prior to assay). The TmNPs are then added 2 days later for a 24 h incubation prior to clonogenic assay or slide staining. For combinations of IUdR and MTX (IUdR+MTX), both drugs are added at the same time the day after cell seeding, for two doubling times of incubation.

### 2.6. Conventional Cell Irradiation Setup

The irradiation of 9LGS cells using conventional broadbeam (CBB) X-rays was performed at the Prince of Wales Hospital, Randwick, Sydney, NSW, Australia, following the irradiation protocol of Oktaria et al. and Engels et al. [7,39,44]. Using a Nucletron Oldelft Therapax DXT 300 Series 3 Orthovoltage unit (Nucletron B.V., Veenendaal, The Netherlands) [7,39,44], this 150 kVp kilovoltage peak energy (66 keV mean energy) [7,39] was chosen to target the maximum mass energy absorption of thulium oxide relative to water [7]. Accordingly, this same peak energy was chosen for IUdR irradiation for the same reason, with the greatest absorption relative to water found for the same inherent filtration most appropriately matching that of iodine (Figure A2). The data for these spectra were produced from XMuDat and sourced from Boone and Chavez, 1996 [49,50].

Monolayers of 9LGS cells were irradiated (at 0.75 Gy/min) in horizontal T12.5 cm^2^ flasks under 6 mm of c-DMEM 50 cm from the source at the surface of the flask in full scatter conditions [7,39,44]. With an inherent filtration of 3 mm Be and an additional 0.35 mm of copper and 1.5 mm of aluminium (HVL = 0.68 mm Cu), the nominal dose rate was 0.75 Gy min^−1^ at the entrance, for a current of 20 mA [7,39].

### 2.7. Synchrotron Radiation Beam Configurations

The irradiation of cell samples was conducted in the Imaging and Medical Beamline (IMBL) of the Australian Synchrotron, Clayton, Melbourne, VIC, Australia, using the dynamic option of IMBL’s hutch 2B. Technical data are summarised in Table A1.

The synchrotron wiggler field was chosen to be 2 T, with Cu/Al filtration (69 keV mean energy) for SBB and MRT for comparable energy to CBB, yet at several hundred times higher dose rates. Ultra-high dose rates of 74.1 Gy/s for SBB and 50.3 Gy/s for MRT peaks (valleys are 5.7 Gy/s) were used. For MRT fields, the ratio of the dose delivered into the peak regions where the X-ray microbeams are incident upon the cells, D_peak_ (Gy), to the dose delivered by the MRT field into the valley spacings between the beam tracks, D_valley_ (Gy), is defined as the peak-to-valley dose ratio (PVDR), as shown in Equation (1).

(1) $$PVDR=\frac{D_{peak}}{D_{valley}}$$

For this experiment, the PVDR was measured as 8.9. As such, all MRT doses referred to in this study are the prescribed valley dose for that MRT field, with the microbeam peaks experiencing an 8.9 times higher dose.

These microbeams were produced by passing the synchrotron beam through a tungsten carbide multi-slit collimator (MSC) that was 8 mm thick, 40 mm wide, and 4 mm high, as described by Stevenson et al. [51]. This produced an array of 25 microbeams at a width of 50 µm and a pitch of 400 µm. The intrinsic irradiation field size of 10 mm × 60 mm used for both SBB and MRT necessitated the use of four columns to irradiate much larger T12.5 cm^2^ flasks and imaging micro slides, translating them vertically in the beam (each filled with Gibco HBSS buffer with phenyl red (ThermoFisher Scientific, Brisbane, QLD, Australia, #24020117)). Details for beam configuration parameters in vitro can be found in Table A1 and further in Dipuglia et al. [26].

### 2.8. Clonogenic Assay

Clonogenic assays were performed for each treatment type to assess long-term cell survival as the radiobiological endpoint as a measure of treatment efficacy [7,52,53]. Following the treatment of cells seeded into T12.5 cm^2^ flasks, 9LGS cell subcultures were harvested and plated at low densities in 10 cm diameter Petri dishes (Corning Primaria™ 100 mm Cell Culture [petri] Dishes; Corning Incorporated, Corning, NY, USA, #353803) to determine clonogenic survival.

After 15 doubling times of incubation, cells were rinsed with Gibco DPBS (with Ca^2+^/Mg^2+^ salts; ThermoFisher Scientific, Brisbane, QLD, Australia, #14040), fixed with 70% ethanol (*v*/*v*), and stained using crystal violet solution (Sigma Aldrich via Merck Life Science, Melbourne, VIC, Australia, #Ht90132) diluted 1:3 in 70% ethanol (*v*/*v*). Colonies with less than 50 cells were discounted, and plates with less than 50 colonies or more than 300 colonies were discounted.

The surviving fraction (SF) is calculated by taking the ratio of the plating efficiency (PE) (the ratio of the surviving colonies counted to the cells seeded at the time of plating) of the treated cells and dividing it by that of the cell-only control for the case of non-irradiated (0 Gy) measurements. When the samples were irradiated, the SF was calculated by taking the PE of the irradiated cells and dividing by the PE of the respective non-irradiated sample.

### 2.9. Confocal Microscopy for γH2AX Imaging

Double-strand DNA breaks (DSBs) were visually revealed via γ-H2AX detection and imaging using confocal microscopy [54,55]. Images were quantified using a subsequent analysis with ImageJ. Microscopy was performed for a monolayer of 9LGS cultured for a confluence of 100,000 cells in the wells of an 8-well micro-chamber (Ibidi) slide in accordance with the abovementioned methods. One well was seeded for each treatment type, and at a confluence of 100%, the cells were irradiated in accordance with the aforementioned configurations and methods; slide wells were also filled with HBSS buffer.

At 20 min following irradiation, cells were washed twice with 300 µL of ice-cold DPBS per well before being fixed with 300 µL of ice-cold 100% methanol per well for 20 min on ice. The wells were then each washed three times with 300 µL of cold DBPS, and for each wash, the chambers were rocked for 5 min at room temperature. Following this, the chambers were treated twice with a blocking solution of 3% bovine serum albumin (BSA; Sigma-Aldrich via Merck Life Science, Melbourne, VIC, Australia, #A9418) in DBPS (BSA-DPBS), with 15 min of rocking at room temperature for each wash. A primary antibody (mouse anti-phospho-histone H2A.X (Ser139), clone JBW301, supplied via Merck Millipore (Merck Life Science, Melbourne, VIC, AUS, #05-636) was added at a ratio of 1:500 in 1% BSA-DPBS, for a concentration of 2 µg/mL, to the cells, which were incubated for 2 h at room temperature.

Following incubation, the cells were washed three times with BSA-DPBS, with 5 min of washing at room temperature per wash. A secondary antibody (goat anti-mouse IgG1 cross-absorbed, Alexa Fluor 488, supplied via Invitrogen (Merck Life Science, Melbourne, VIC, Australia, #A21121) was added at a ratio of 1:500 in 1% BSA DPBS, for a concentration of 4 µg/mL, to the cells and incubated at room temperature for 1 h in darkness. Finally, the cells were again washed twice with 300 µL of DBPS before 100 µL of DBPS was added to each well. A total of 2 µL of 1 mg/mL Hoechst 33342 (Sigma-Aldrich via Merck Life Science, Melbourne, VIC, Australia, #14533) was then added to each well for 20 min at room temperature before cells were imaged with a Leica TCS SP8 confocal microscope (Leica, Microsystems, Wetzlar, Germany) with a 93× glycerol objective at room temperature.

The confocal microscope utilised a laser, providing a 488 nm excitation with a detection range for the Alex Fluor 488 fluorophore (FITC), and another 405 nm laser providing the range for the Hoechst 33342 nuclear counterstain (DAPI). Detection ranges were set to a minimum of 10 nm above the excitation wavelengths for each laser and higher. A 2 × 2 tile scan with a z-stack of 10 slices was taken per image. These images were then analysed via the Lecia LasX Application Suite (v. 3.0.11.20652, Leica, Microsystems, Wetzlar, Germany), ImageJ (v. 1.53k; NIH, Bethesda, MD, USA) [56], and Microsoft Excel (v. 2016; Redmond, WA, USA).

### 2.10. Image Processing and Analysis

For the quantification of the total enhancement in DSBs observed in a γH2AX image, a quantitative analysis of γH2AX foci (an object representing a nuclear DSB site) was used as the key indicator of DNA damage due to the high sensitivity of this method [54,55]. For this study, the foci factor (FF) method was used to account for variations in individual γH2AX foci. This method follows our previous work establishing this method in Valceski et al. [30]. Following this method, the DSB enhancement ratio (DSBER) discussed in the results was determined as the ratio of foci factors of a treatment sample to the untreated 0 Gy control (Equation (2)).

(2) $$\frac{FF_{treatment}}{FF_{control}}=\frac{DSB_{treatment}}{DSB_{control}}=DSBER$$

This DSBER value is used as the final quantification of all confocal images using the γH2AX assay in this study and represents a quantification of the enhancement in DSB induction in a sample.

A comparison of the mechanisms of synchrotron MRT was also made possible via the quantitative analysis of both 93× and 20× resolution γH2AX images. Using ImageJ [56] (version 1.53k) to draw regions of interest (ROI) around peaks and valleys separately in 93× images, foci data could be collected and analysed to determine FF values and average DSB enhancement ratios across at least 6 replicate images in both peaks and valleys separately. This average was normalised to that of the 0 Gy cell-only control to obtain a DSBER value for each peak and each valley across all treatments. This analysis was performed for both 0.5 Gy and 5 Gy valley doses to allow for comparisons between equivalent doses, including 5 Gy CBB and SBB.

This result further allowed for a biological peak-to-valley DNA damage ratio (BPVDR) to be acquired, which is expressed in terms of the FFs (Equation (3)) for separate peaks and valleys, to compare the biological effects to the physical dosimetry (measured in the PVDR).

(3) $$BPVDR=\frac{FF_{peak,treatment}}{FF_{valley,treatment}}$$

An additional analysis also included the sectioning of independent replicates of 20x images into 16 ROIs of MRT peaks. ImageJ permitted intensity data from 2D plot histograms to be extracted to unveil the shape and size of the MRT peaks, as expressed in γH2AX foci intensity along the x-axis of the image (intensity values of all pixels along the y-axis were averaged to acquire the intensity profile). This allowed for data from 16 beam segments to be averaged to determine an overall 2D plot profile of the beam. These data are expressed as a histogram showing intensity as a percentage of the average peak (of the MRT peak) intensity vs. the off-axis lateral distance in microns. The widths of the peak in terms of the full width at half-maximum (FWHM) were then found.

### 2.11. Statistical Analyses

All error bars were calculated as the standard error of the mean (SEM) using 2 standard deviations (95% confidence interval) of the mean divided by the square root of the number of images used (i.e., FF values being averaged). For all samples tested, at least 6 replicates (n = 6; images and plates) across independent repeats were averaged for each sample.

## 3. Results

### 3.1. Synchrotron Broadbeams and MRT Increase Cancer Killing When Enhanced with Radiosensitisers

**Figure 1:**
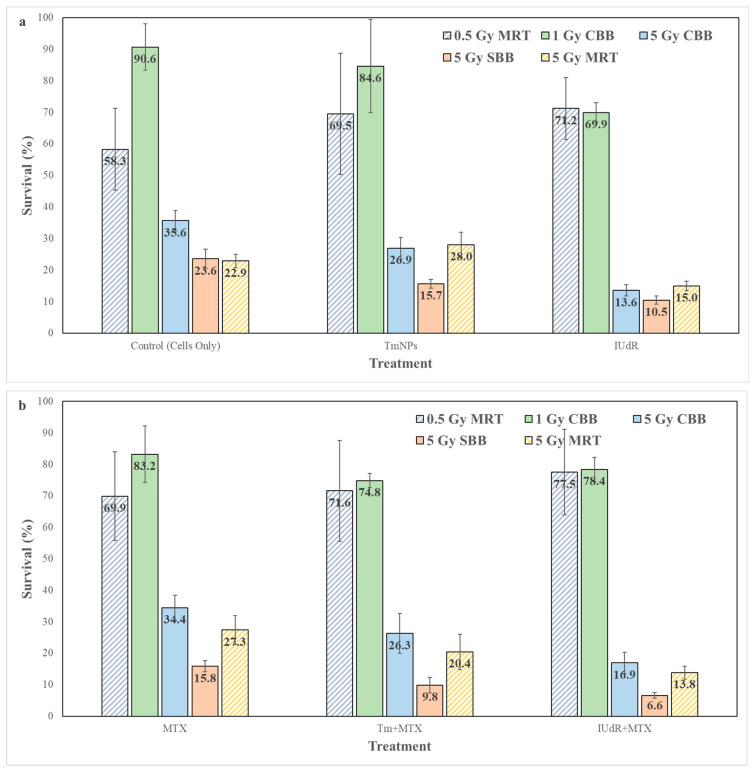
Cell survival for various treatments and radiation fields. Clonogenic assay reveals the long-term cell survival of 9LGS in response to (**a**) various radiosensitisers (TmNPs and IUdR) and (**b**) combinations with MTX (Tm+MTX, IUdR+MTX) when irradiated. RT modalities include conventional broadbeam (CBB) orthovoltage X-rays (at lower (solid green) and higher (solid blue) dose fractions), compared with synchrotron broadbeam (SBB) X-rays (solid red) and microbeam radiation therapy (MRT), with lower and higher dose fields of 0.5 Gy (striped blue) and 5 Gy (striped yellow) (MRT doses listed are the prescribed valley doses, whereas the peak doses are PVDR = 8.9 times greater). Error bars represent the standard error of the mean (SEM) (using standard deviations at the 95% confidence interval). An average of 6 replicate samples (n = 6) is used across independent repeats.

A clonogenic assay measured cell survival in Figure 1, in which radiation-only MRT enhanced cancer killing compared with CBB X-rays at the same 5 Gy dose. Figure 1 also demonstrated that TmNPs and IUdR radiosensitisers yield a decrease in cell survival, in which DNA-localised IUdR induced far greater cancer killing than cytoplasm-localised TmNPs. This phenomenon has been previously observed in studies comparing NPs with halogenated pyrimidines [39]. This effect highlights the importance of proximity to DNA, where NP enhancement and improved cell death resulted from a greater incidence of DSBs due to secondary electron emissions from NPs [7,57].

Similar enhancement is seen with SBB X-rays, where the UHDR at 74.1 Gy/s is observed to kill more 9LGS than with CBB X-rays at 0.0125 Gy/s, all whilst maintaining the cancer-killing provided by TmNPs and IUdR (Figure 1). Whilst CBB does not result in enhanced 9LGS killing with MTX (alone or combined), MTX provides significant enhancement with SBB X-rays, resulting in cell survival falling to half of that of CBB at the same 5 Gy dose (Figure 1).

**Figure A1:**
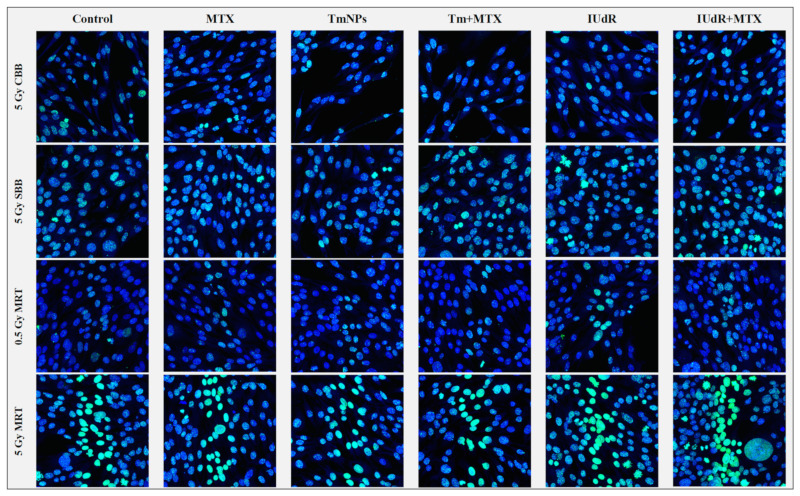
γH2AX image panel of 9LGS with various treatments and radiation fields. Synchrotron broadbeam (SBB) and conventional broadbeam (CBB) orthovoltage X-rays at 5 Gy for the cell-only control (Control) and each radiosensitiser agent and combination are compared with MRT fields with 0.5 Gy and 5 Gy prescribed valley doses (peaks at PVDR = 8.9 times the valley dose). Using a confocal microscope at 93× resolution, the green channel representing γH2AX foci (correlating with DSBs) is overlayed on the blue channel representing 9LGS cell nuclei with a DAPI nuclear counterstain.

**Figure A2:**
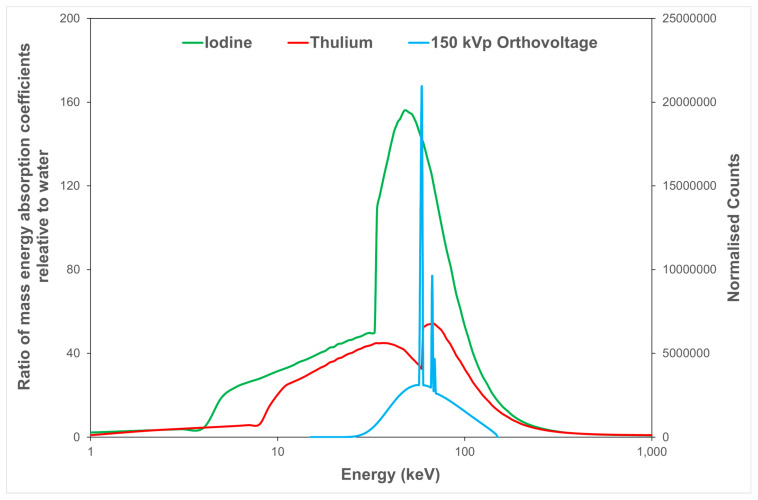
Overlay of the ratios of the mass energy absorption coefficient of radiosensitiser elements relative to water. Both thulium and iodine coefficients of µ_en_/ρ for total mass energy absorption are shown normalised relative to water with the 150 kVp photon energy spectrum for the Nucletron Oldelft Therapax DXT 300 Series 3 Orthovoltage unit (Nucletron B.V., Veenendaal, The Netherlands) at the Prince of Wales Hospital, Randwick, Sydney, NSW, Australia. The data for these spectra were produced using XMuDat and SpekCalc and sourced from Boone and Chavez, 1996 [49,50,71].

This result not only affirms greater cancer cell-killing with UHDR X-rays but radiosensitisation with MTX present. Our previous work demonstrates that overwhelming DSBs can be induced by both UHDR radiation and radiosensitising agents (Figure A1) [30]. When agents like MTX are used to enhance cell-killing, DNA repair mechanisms may be saturated, and SSBs may be unable to repair themselves, so they convert to DSBs over time [16,58,59,60,61,62,63]. MTX is known to inhibit DNA replication and thus cell and DNA growth and repair [44,46], which further exacerbates this effect and results in greater DNA damage (Figure A1) and therefore greater cell-killing (Figure 1).

**Figure 2:**
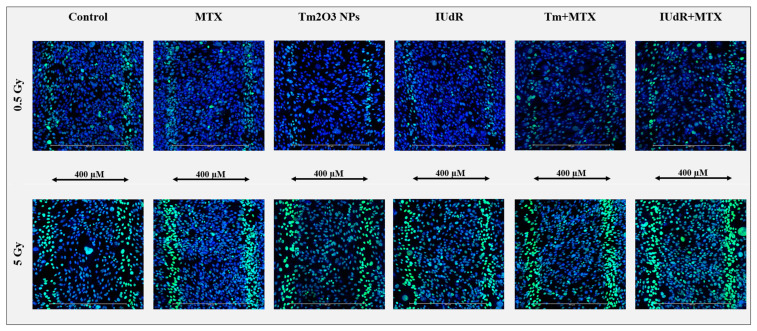
γH2AX immunofluorescent confocal microscopy comparing multiple MRT peaks for various radiosensitisers. Panel displays 20x dry resolution images of 9LGS cells as the maximum projection of 5-slice Z-stacks. All images are overlays of two channels, with double-strand DNA breaks (DSBs) represented by green γH2AX foci overlayed on a Hoechst 33342 nuclear counterstain (blue). Radiosensitiser treatments are displayed in columns across the panel and show DSB changes 20 min after irradiation began for all cases in 9LGS cells. Radiation treatments change down the two rows to 0.5 Gy (valley dose) synchrotron MRT (**top**) and 5 Gy (valley dose) MRT (**bottom**). PVDR = 8.9 for all. Images are representative of a set acquired for each sample time. Each sample that was imaged was fixed at 20 min post-irradiation.

MRT survival is shown to be potentially dominated by high levels of cell death at the peak, suggesting that it is a driver of cancer cell-killing [22]. Figure 1 demonstrates this, with a 0.5 Gy (valley dose) MRT field, where most of the radiation is concentrated in the peak, and 9LGS cell survival is equivalent to or lower than that of 1 Gy CBB X-rays for all treatments. Given that a peak only occupies 1/8 of the MRT field, this demonstrates the significant damage to cells induced by MRT peaks, considering that a CBB field applies the same 1 Gy dose to the entire field for the same or greater survival. Indeed, as Figure 1 indicates that a 0.5 Gy MRT peak can kill as much 9LGS as a CBB field with a several times higher dose, Figure 2 (and Figure A1) consequently demonstrates that this results from overwhelming DSBs in peaks.

While 5 Gy MRT shows equivalent killing to SBB (indicating its tissue-sparing potential), MRT also demonstrates enhancement with radiosensitisers. The greatest high-Z enhancement occurs with IUdR and IUdR+MTX, indicating the importance of DNA localisation and demonstrating the radiosensitiser enhancement potential of MRT in delivering greater RT cancer efficacy. This local dose enhancement bodes well for potential usage in improving MRT cancer-killing and thereby permitting lower dose fractions to be used to spare tissue whilst maintaining tumour control (a key objective of UHDR RT and MRT). To further explore the mechanisms of these radiosensitiser enhancements and improve our understanding of MRT, DNA damage following treatment was then investigated.

### 3.2. DNA Damage Imaging Reliably Affirms MRT Field Collimation and Changes with Radiosensitisers Present

Confocal images at 20× resolution provided spatial information on MRT biological effects by visualising the beam (through cells fixed at 20 min post-irradiation) in terms of DSBs represented by γH2AX foci. Figure 2 demonstrates the accuracy of the physical dosimetry, whereby for 0.5 Gy and 5 Gy (valley dose) MRT fields, a 400 µm microbeam spacing (peak mid-point to mid-point) was verifiable in each image for every sample type due to the high sensitivity of the assay (1.2 mGy is the minimum detectable dose) [54]. Upon observation, all peaks are also approximately the same size, with their widths measured in tens of microns and the prescribed width dosimetrically set at 50 µm.

However, while Figure 2 shows microbeam tracks to be quite consistent in width for all 0.5 Gy images, the 5 Gy MRTs are noticeably wider, with additional DNA damage spilling out into the surrounding valleys. This demonstrates that the higher dose has contributed to a greater scatter (or roll-off) of radiation dose into the valley. This is notably true in the near-beam transition zones, in which Schültke et al. describe Compton scatter and secondary electrons as providing additional doses to cells above that of the nearby valley but below that of the peak [25]. Given a PVDR of 8.9, the peak dose for cells in the beam tracks would be over 44.5 Gy. This large amount of radiation will scatter significantly more into the valley compared to the 0.5 Gy (valley) MRT field, hence the higher dose in 5 Gy fields, where more of the dose is scattered; therefore greater DSBs are induced in these valleys (Figure 2 and Figure A1).

**Figure 3:**
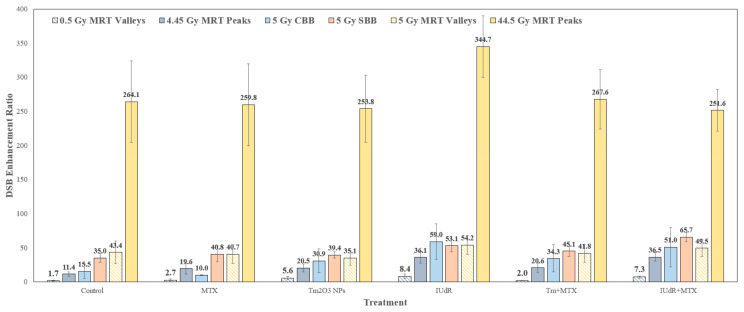
Quantification of γH2AX confocal images broken into peak and valley regions of interest. Images with 93× resolution are used to quantify DNA damage in treatments across peaks and valleys separately, compared with CBB and SBB fields. Results yielded DSB enhancement ratios for both 0.5 Gy and 5 Gy valleys and their respective peaks (PVDR = 8.9) relative to 0 Gy cells only. All results are expressed in terms of the ratio of FF of each treatment normalised to the FF of the 0 Gy control. Error bars represent the standard error of the mean (using standard deviations at the 95% confidence interval). Each collection of images for each treatment and radiation regimen was analysed to obtain an average FF across at least 6 replicate images for each treatment type for 0.5 Gy and 5 Gy MRT field fractions (MRT doses listed are the prescribed valley doses). All data points displayed in this figure represent the average of at least six quantified images across independent experimental trials (n = 6) and display error bars representing the standard error of the mean (SEM). Each sample that was imaged was fixed at 20 min post-irradiation.

Other studies have also observed higher foci densities resulting from nearby NP clusters in the cytoplasm surrounding the nucleus, which correlates with the reduced cell survival seen in Figure 1 [7,30]. Additionally, a greater incidence of potential cell death is observed (indicated by fragmented DNA and morphological cell shrinkage or swelling), as well as only half a cell irradiated by MRT peaks when caught in the transition zones at the edges [63,64,65,66]. These are observable in Figure 2 (and Figure A1), with radiation roll-off from peaks into valleys that appear to slightly widen the peaks, compared to 0.5 Gy MRT (Figure 2). This is also observable with IUdR and IUdR+MTX particularly, whereby the most numerous and luminous foci are visible in Figure 2, notably in the valleys. This correlates well with Figure 1, demonstrating dose enhancement and improved cancer-killing with these radiosensitiser treatments, and further correlates with minor increases in DSB levels in Figure 3.

### 3.3. MRT Peaks Produce Consistent Overwhelming Levels of DNA Damage, While Valleys Are Comparable to Broadbeams and Show Radiosensitiser Enhancement

A breakdown of DSB enhancement in peaks and valleys was obtained using Equation (2). The result in Figure 3 demonstrates several important biophysical features of MRT. The high dose delivered in the 44.5 Gy peaks appears to overwhelm 9LGS cells with DSBs, such that no additional DSB enhancement is seen in peaks across radiosensitiser treatments. This is the case for all radiosensitisers, albeit with a minor increase in DSBER for IUdR-treated cells (however, this is within the margin of error of the other treatments at this dose). This may also suggest that mechanisms other than DSBs (such as DNA repair saturation and SSB conversion to DSBs [16,58,59,60,61,62,63]) are responsible for the reduced cell survival observed with SBB compared to MRT (Figure 1), given the apparent overwhelming quantity of DSBs induced with UHDR SBB X-rays compared to CBB (Figure 3).

This is further correlated by comparing SBB and MRT valleys at the same 5 Gy dose. Figure 3 shows an important relation, whereby the radiobiological effect of MRT valleys and SBB are equivalent at the same dose, such that the same enhancement in DSBs is seen, including with different radiosensitisers. This correlates with previous theories in several MRT studies, including confirming findings by Ibrahim et al., who also found biological equivalence with MRT valleys and broadbeams [22,67,68]. A novel finding is that this equivalence is observed regardless of which radiosensitiser combinations are used, or even which dose rate, despite SBB fields having approximately thirteen times the dose rate of MRT valleys (Table A1). The fact that these two different fields used the same dose is enough to induce the same quantity of DSBs. It is then proposed that Figure 3 demonstrates equivalence in the biological effects of SBB and MRT valleys.

However, as previously discussed, the increased cell-killing found in Figure 1, with SBB over MRT, highlights the need to consider DNA SSBs as an alternative key mechanism of cancer-killing in cells treated with high-dose rate radiotherapies. Engels et al. found an increased probability of SSBs (via the β component of the linear quadratic model) in 9LGS cells with 2 T Cu/Al (71 keV) SBB X-rays [22]. Figure 2 and Figure 3 then highlight the potential role that SSBs play in inducing the additional DNA damage to cells (in response to UHDR effects) that is needed to induce significant tumour cell-killing (Figure 1). This is also possible given that SBB and MRT valleys (which comprise most of the MRT field) have equivalent DSBs at the same dose (regardless of radiosensitiser), yet SBB kills significantly more 9LGS cells in the long term (Figure 1). It is also worth noting that reductions in clonogenic survival may also be explained by deficient repair mechanisms [69]. In this case, more complex DSB sites (which are possible with MRT peaks given the overwhelming damage observed in Figure 2 and Figure 3) or chemotherapeutic drugs (notable with MTX drugs [44,46] and SBB X-rays in Figure 1) may impair these repair processes, further resulting in increased cell-killing at the 5 Gy dose used.

For lower doses, 4.45 Gy MRT peaks are not observed to be equivalent to 5 Gy SBB (Figure 3). The radiation-only control for this MRT field exhibits a DSBER value comparable to that of 5 Gy CBB (Figure 3), and while other radiosensitiser treatments are higher than this, they are mostly comparable to their conventional counterparts. There is still a significant increase in DSBs that is close to but not quite at the level at which peaks and SBBs are equivalent. This may be due to the 4.45 Gy peaks giving a more than 10% lower dose than 5 Gy SBB and MRT valleys or possible tissue-sparing intrinsic to MRT (which is delivered primarily by its peaks), which Ibrahim et al. alluded to in suggesting that MRT alters cell behaviours while SBB does not [67]. It is notable, however, that there was still a significant increase in DSBs induced by IUdR and IUdR+MTX radiosensitisers in these low-dose MRT peaks. Additionally, IUdR and IUdR+MTX increase DSB incidence in MRT valleys (and broadbeams) in Figure 3 (at both doses given), which is correlated with Figure 1 for 5 Gy MRT fields that demonstrated MRT dose enhancement with these radiosensitisers. This further correlates with radiosensitiser enhancements from these DNA-localised high-Z treatments in Figure 1, which delivered enhanced cancer killing in MRT. This still bodes well for the use of radiosensitisers to enhance MRT efficacy in cancer treatment and suggests that while peaks drive cancer-killing, further enhancement can be delivered by radiosensitisers in valleys. This also means that a lower MRT dose could be given in a fraction with these high-Z agents present whilst maintaining tumour control and treatment efficacy. This can improve normal tissue-sparing potential due to reduced exposure.

Moreover, given that a third of the cell population was killed by low-dose MRT in Figure 1, this would also support the hypothesis proposed previously. The peak is the cause of most cell death in MRT fields, with the remaining enhancement arising in valleys (given that all treatments with 0.5 Gy MRT demonstrated less 9LGS survival than 1 Gy CBB despite delivering a lower dose to much of the field). It is also likely that the DNA damage induced in tumour cells is more dependent on the radiation field geometry and dose given rather than the dose rate, given that microbeam peaks and broadbeams experience similar rates. With both of these dose rates well above the 40 Gy/s intrinsic threshold for UHDR effects [19,21], it is clear from Figure 3 that similar dose rates do not necessarily yield equivalent radiobiological effects in cancer cells despite similar collimations or prescribed doses. Instead, it is proposed that MRT peaks exhibit different effects altogether in living cells at the same dose and UHDR, especially with radiosensitisers present. This was evident in an analysis of MRT peaks, in which radiosensitising high-Z NPs altered the geometry of these regions of the MRT field.

### 3.4. Bio-Dosimetric Analysis of MRT Peaks Reveals Peak Broadening with Nanoparticles

**Figure 4:**
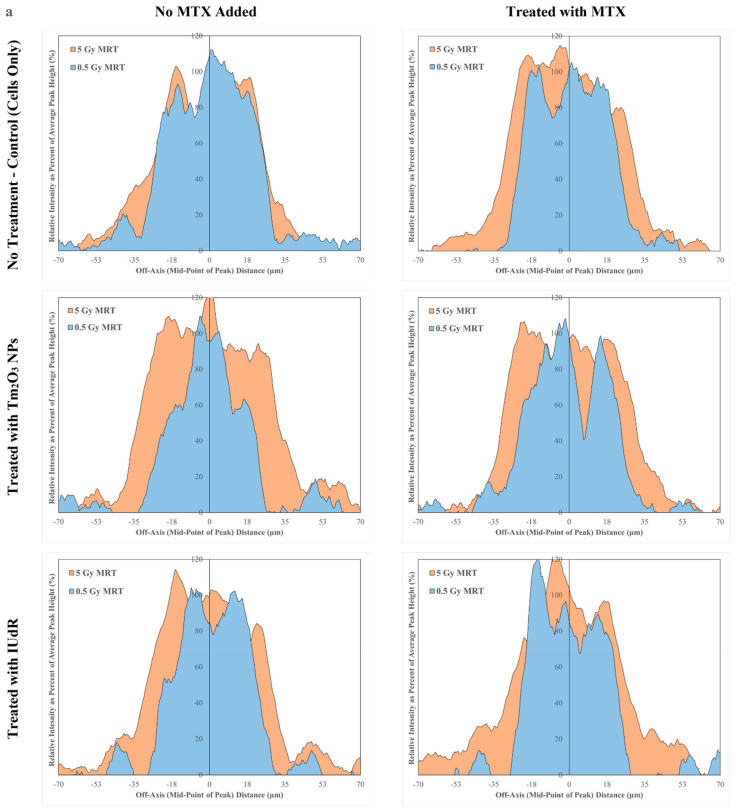

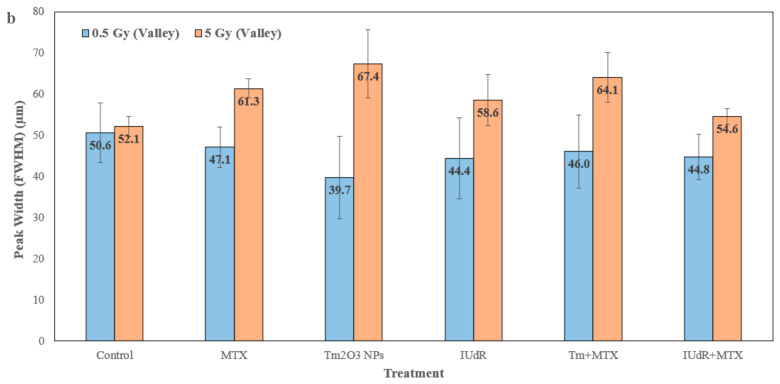
MRT beam profiles and peak widths expressed via γH2AX foci. (**a**) Beam profiles from 20× dry confocal microscopy images of MRT peaks for each treatment at both 0.5 Gy (blue) and 5 Gy (orange) (MRT doses listed are the prescribed valley doses) are shown to reveal peak broadening, with the left column showing MTX-negative treatments and the right showing MTX-positive treatments. These data are expressed as a histogram showing intensity as a percentage of the average peak (of the MRT peak) intensity vs. the off-axis lateral distance in microns. Radiation-only MRT is shown in the top row, followed by TmNP treatments in the middle and IUdR treatments at the bottom. (**b**) Peak widths are shown to quantify broadening results in subfigure (**a**) and are the average of the profile data across 16 peak segment ROIs taken across independent repeats.

An MRT peak analysis of 20x γH2AX images was performed to extract 2D histogram profiles of the MRT beams. These plots unveiled the shape and size of the MRT peaks expressed in γH2AX foci intensity along the x-axis of the image (Figure 4a). This permitted a radiobiological analysis of MRT beams compared to their physical expectations, track uniformity, and radiation scatter into surrounding valleys. Peak width measurements revealed that the radiobiological effect of MRT, combined with radiosensitisers at higher doses, induces a broadening of the peak (Figure 4a), thereby increasing the radiation dose to cancer cells in the nearby valley. The widths of the peak expressed as full width at half-maximum (FWHM) were then found (Figure 4b).

The 0.5 Gy MRT was not observed to induce any notable change in the width of peaks for any treatment (Figure 4a). The width of the MRT peak at this dose remained consistent for all radiosensitisers (and within statistical error) in Figure 4b. Peak broadening was most notably observed for 44.5 Gy peaks with treated samples. The MRT control peak remained at 50 µm, within the error of the width of the lower-dose 4.45 Gy peak, as well (Figure 4b), yet it still demonstrated some roll-off of radiation-induced DSBs into the nearby valley (Figure 4a). The 44.5 Gy dose of the peak sees an even larger roll-off into the near-beam regions of the valley when 9LGS cells are treated with radiosensitisers in conjunction with a broadening of the peak.

The largest peak broadening was observed in cells treated with TmNPs, with or without MTX. Figure 4b demonstrates peaks with TmNPs present broadened to as large as 67 µm, with comparable peak widths of 64 µm for 9LGS cells treated with both TmNPs and MTX. This broadening represents 34% and 28% broadening, respectively, demonstrating that the large number of secondary electrons produced by the high dose to the peak allows for peak-equivalent DSBs to be induced in the valley transition zones.

While peak broadening was not observed for the 0.5 Gy MRT field, even for TmNPs, it can be noted that the TmNP peak profiles exhibit small extensions at the edge of the peak, with a short plateau and broadening at off-axis distances of approximately 15–30 µm from peak centre (Figure 4a). This demonstrates that around the edge of the beam and into the immediate near-peak regions of the valley, a notable increase in DSB induction takes place. Engels et al. found a significant increase in the dose into cells at very similar distances out from the beam centre, albeit with tantalum oxide NPs, another high-Z nanoparticle, using Geant4 simulations for a 70 keV SBB X-ray beam (compared to 71.4 keV used in this study) [38]. Tantalum NPs (Z = 73) and TmNPs (Z = 69) have similar Z values and have been previously found to have very similar radiosensitisation potential across multiple beam energies [7,39]. It was also found that these occur with tantalum forming shell-like clusters around the nuclei of 9LGS cells, which is an observation similar to findings in previous studies that also demonstrate TmNP clusters in 9LGS cells gathering near nuclei [7,30,38].

These findings highlight that TmNPs also exhibit these simulated properties, with increased doses at the peak edge producing additional DSBs due to the NPs. Figure 4a also shows that these TmNP low-dose effects only induce DSBs at approximately half the intensity of the peak, suggesting less dose and DNA damage in these peak-like extensions. It can be hypothesized that the secondary electrons emitted by the present NPs produce peak extensions at the beam edge due to internalised NP clusters present. It is possible, given that the high peak doses generate significant scatter into the valley, notably at the edges of the beam tracks and in the immediate near-beam regions [25], that this will activate these NPs. Subsequent photon absorption would result in secondary electron production producing substantial DSBs in 9LGS cells in these regions (Figure 4a), with DNA damage levels comparable to the peak itself due to this ‘splash’ of extra dose into these transition zones. This may also help to explain the overwhelming DSBs in 9LGS with the 5 Gy MRT fractions observed in Figure 2 (and Figure A1).

While the physical collimator permits only 50 µm-thick beam tracks, Figure 4 highlights that the biological effect of these high-dose peaks can be extended beyond this into the valley, permitting significant DNA damage to valley cells beyond the peak. This result correlates well with 9LGS survival in Figure 1. MRT survival for 5 Gy fields has not been observed to induce a dose rate effect; however, 9LGS survival is as low or lower than the 5 Gy CBB equivalent (Figure 1). Whilst only 12.5% of the 9LGS cell population would experience the full high-dose rate peak with MRT radiation-only fields, samples pre-treated with TmNPs should experience peak coverage and cell death of nearly 17% of the population due to the peak broadening seen in Figure 4a. This biological broadening effect may help to explain any additional killing that is observed. This may also be useful in treating cancer cells with cancer-specific NPs, as this effect could have the potential to induce peak broadening in cancer cells whilst maintaining the healthy tissue-sparing of MRT.

### 3.5. Biological PVDR Matches the Physical and Reduces Due to Radiosensitiser Enhancement in Valleys

**Figure 5:**
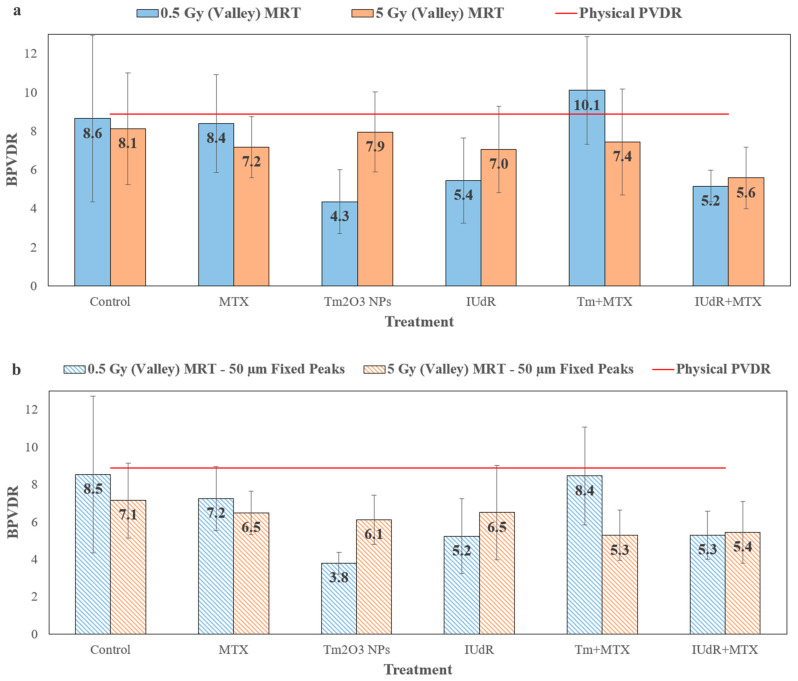
Biological PVDR values (BPVDR) for all treatments. MRT fields at 0.5 Gy (blue) and 5 Gy (orange) doses (MRT doses listed are the prescribed valley doses) compare the biological effect of MRT peak vs. valley doses relative to the physical dosimetry. Quantitative analysis of 93x resolution γH2AX confocal images broken into peak and valley regions of interest (ROIs) allowed DSB FF factors to be obtained for peaks and valleys separately and then allowed the ratio of peak to valley to be found. All BPVDR (Equation (3)) values are taken as the average of ratios found individually for at least 6 replicate images. The red line in each graph represents the physical PVDR of 8.9. (**a**) BPVDR is shown for the case in which the broadening of the peak is considered. (**b**) BPVDR for the cases in which the peak width changes are not considered, and ROIs are fixed at 50 µm in width and centred to align with the image peak.

The FF values for each peak and valley could also be used to obtain a biological peak-to-valley damage ratio (BPVDR), expressing the difference in peak vs. valley DNA damage in terms of DSBs. Whilst previous studies have encountered some difficulties in determining BPVDR values [28,29], this study has found success in matching BPVDR to PVDR for MRT (Figure 5). This BPVDR (Equation (3)) allows for a direct biological comparison between DSBs (which are directly proportional to dose under the linear quadratic model) [69] and the physical PVDR to verify any differences. BPVDR values are shown in Figure 5, which includes values that account for peak broadening and scatter effects seen in Figure 4, as well as values that do not consider peak broadening and use ROIs centred on peaks at a fixed width of 50 µm.

The BPVDR values in Figure 5 reaffirm the peak broadening effects seen in Figure 4 and demonstrate the radiobiological effects of MRT as experienced by cells. Figure 5a accounts for peak broadening and therefore demonstrates the real biological changes, as expressed in DSBs, between peaks and valleys in the 20 min following MRT. Figure 5 demonstrates that the BPDVR values for MRT radiation only match the physical PVDR, falling well within experimental error. This result demonstrates a direct relationship between the physical dose delivered and DSBs induced in 9LGS cancer cells. This is notably due to the value of the microscopy assay used in providing spatial information that allows peaks and valleys to be distinguished [28] and also due to its sensitivity [54,55].

However, all radiosensitiser treatments or dose BPVDRs are lower than the prescribed physical PVDR of 8.9 (as is the 0.5 Gy MRT, although it is not significant as it is within experimental error). Some studies have suggested that the overwhelming damage to MRT peaks may result in foci saturation [29], yielding less peak damage when analysed and thus reducing BPVDR. However, we did not encounter saturation issues with γH2AX images in this study, and we were able to successfully analyse all the images (Figure 3 and Figure A1).

Whilst Rothkamm et al. suggest that slight decreases in BPVDR may be due to limited cell movement in the culture [28], we propose that our result indicates that additional DSBs and biological effects are induced in valleys. This then increases the DNA damage induced within the 20 min following MRT. This would be reflected in a reduction in BDPVR below the prescribed PVDR, as the valley would express additional DSBs arising from the extra damage induced by the radiosensitisers. Additionally, Figure 3 showed the expected increases with present radiosensitisers in 4.45 Gy peaks, suggesting that enhancement in peaks is still present. However, due to the far greater field coverage of the valley, the valley damage dominates and thus has a greater effect on BPVDR, hence reducing the value. This observation would indicate the importance of valleys as the more dominant dosimetric factor in MRT treatment planning.

Figure 5b follows these trends but also demonstrates even lower BPVDR than Figure 5b, considering that it does not account for peak broadening. This is due to Figure 5b reflecting both the biological effects in Figure 5a, as well as the peak broadening observed in Figure 4. Wider peaks would roll off additional DSBs into valleys, contributing to far higher valley damage and therefore a lower BPVDR. Figure 5b thus supports the result found in Figure 4.

As Figure 3 demonstrates high levels of 44.5 Gy MRT peak DSBs, any additional enhancement in DSBs is derived from dose enhancement delivered by radiosensitisers in the valleys of those fields at higher dose fractions. Figure 5a highlights this, as all radiosensitisers see a reduction in BPDVR, suggesting that damage to the valleys is enhanced far more than peaks in the presence of these agents. For 0.5 Gy fractions, except for Tm+MTX and MTX treatments, the radiosensitisers generally enhance the valley even more than 5 Gy doses. This may be due to enhancement from high-Z radiosensitisers producing enough DSBs to contrast with the lack of DSBs in the valley due to the very small 0.5 Gy dose. With sufficient dose enhancement and therefore DSB increase in the nearly unirradiated valleys, the BPVDR would decrease. The bystander effect may also be a contributing factor, as these cells in such low-dose valleys are not directly irradiated [70] and barely receive any dose, yet they still demonstrate sizable DSB enhancement (Figure 3 and Figure 5) and more overall cell-killing (Figure 1).

An additional factor may be secondary electron scatter into the nearby valley from the peak, as observed in Figure 4 with TmNPs. Engels et al. found that Geant4 simulations predict a small increase in dose around the peak–valley edges at lower MRT doses [38], which is correlated with the result in Figure 4 in this study. It is hypothesised from these results that the presence of the NPs in 9LGS when irradiated with MRT may induce additional dose enhancement from the peak into the valley through a shower of these secondary electrons, contributing to the increase in valley damage in the near-peak regions of the valley and therefore reducing the BPVDR.

Notably in Figure 5, IUdR+MTX sees a large reduction, with the BPDVR being approximately half the PVDR, highlighting a doubling of damage in the valley compared to what would be expected at a given dose. This correlates with the IUdR+MTX at 5 Gy MRT result in Figure 1 and would explain the notable enhancement seen given that the enhancement in DSBs in the valley would result in far greater 9LGS cell-killing (as the valley occupies most of the MRT field). Overall, Figure 5 demonstrates generally higher DSBs in valleys than PVDR would physically predict, notably when high-Z radiosensitisers are able to provide significant local dose enhancement.

This highlights both the importance of considering that the biological effects of MRT can be notably different to the physical expectation and how radiosensitiser-enhanced MRT can be used to deliver increased damage to cancer cells in the valley spacings between microbeams. With cancer-specific radiosensitisers, it is then possible to combine these multi-modal systems with synergistic ultra-HDR MRT fractions to deliver significant doses to tumours selectively, with resulting radiobiological damage nearing that of the peak across the entirety of the tumour covered by the MRT field. Additionally, it is also then possible to use this methodology to aid in the pre-treatment planning of potential clinical MRT (which is essential given the rise in pre-clinical MRT studies involving substantive treatment planning [68]), as the correlation between BPVDR and physical PVDR could be used to indicate the expected tumour response. Most importantly, reduced BPVDR with radiosensitisers present then indicates the expected local dose enhancement in valleys, which in turn improves tumour response to treatment. This further permits a lower MRT dose to be given whilst maintaining tumour control, which allows for improved MRT tissue-sparing by reducing radiation exposure.

## 4. Discussion

We have highlighted the enhancement of the radiation dose and subsequent DNA damage in brain cancer with additive combinations of Tm+MTX or IUdR+MTX radiosensitisers. The use of UHDR SBB X-rays and MRT was observed to reduce 9LGS survival even further compared to the CBB X-rays, and even more significantly so with prior exposure to MTX. This synergistic effect was observed to result from a greatly increased incidence of DSBs, especially in MRT peaks in which concentrated radiation was observed to induce overwhelming DNA damage.

The heavy damage to 9LGS cells in MRT peaks was indicated to be the primary cancer cell-killer, especially in 5 Gy MRT fields in which peaks were overwhelmed with DSBs. A clonogenic assay revealed that even a 4.45 Gy peak covering a fraction of a radiation field could yield the same survival as CBB fields with multiple times higher doses than the surrounding 0.5 Gy valleys. Most of the MRT field could be exposed to a negligible quantity of radiation, yet the targeted peak region could still be successfully treated. This highlighted the potential tissue-sparing capabilities of MRT, in which peaks are sufficient to sustain MRT cancer control at this dose, hence allowing lower doses (to much of the field compared with CBB) to be used to reduce harmful exposure to healthy tissues. Conversely, radiosensitiser-enhanced MRT can deliver further dose enhancement in valleys, also permitting lower radiation doses to be given whilst achieving the same efficacy (Figure 1). This bodes well for using MRT as a targeted, multi-modal, radio-surgical modality for cancer therapy, in which precision targeting and targeted radiosensitisers can be used to improve treatment accuracy and tissue protection.

The use of γH2AX DSB images for bio-dosimetry also revealed and verified several mechanisms of MRT, including radiosensitiser-enhanced MRT. Equivalence in the biological effect in terms of DSB expression was observed between SBB fields and MRT valleys, correlating with previous theories and works [22,67,68]. This suggests radiobiological and biophysical similarities between the delivery of broadbeam fields and the radiation scatter and dose roll-off from MRT peaks into valleys. The BPVDR values also matched the physical PVDR for MRT radiation-only treatments, indicating the accuracy of the analysis method and the predictable translation of escalated dose into DSBs. The BPVDR reduced when radiosensitisers were added, arising from dose and DNA damage enhancement to valley populations of cancer cells. Additionally, a unique radiosensitiser effect was observed; MRT peaks broadened in the presence of NPs. When TmNPs are used, alone or combined with MTX, MRT peaks, with higher doses increased as much as 30%, elevating the transition zones in the near-peak regions of the valleys to peak-equivalent damage. This effect is proposed to contribute to the additive effects of NPs combined with UHDR radiation.

Most importantly, radiosensitiser-enhanced MRT also produced additive effects when IUdR and IUdR+MTX were used, although this did not reduce cancer cell survival below that of SBB, even when the doses matched. This is despite significantly more dose delivered in the spatially fractionated peaks, which have been verified in this study to generate significantly higher DNA damage to cells caught in these regions of the MRT fields, resulting in greater cell death. The dose rates provided to MRT valleys were well below that of the SBB fields and therefore the expected levels for UHDR effects, which potentially contributed to the lack of enhancement. However, this may indicate the importance of the dose rate in enhancing cancer control. Regardless, this is still an essential finding given that local dose enhancement by IUdR and IUdR+MTX improves tumour control through greater 9LGS cancer-killing. This in turn supports our hypothesis proposed at the beginning of this study, which states that radiosensitiser-enhanced MRT reduces the BPVDR through local valley dose enhancement and thus improves cancer-killing. This allows a reduced MRT dose to be used for treatment whilst tumour control is maintained, thereby improving and reducing normal tissue radiation exposure risks when targeted radiosensitisers are used.

## 5. Conclusions

This study has produced several mechanistic findings to improve our understanding of the cancer treatment capabilities of radiosensitiser-enhanced UHDR radiotherapy and MRT. Additional and higher dose rates could be explored to further deepen this understanding of the mechanisms and bio-dosimetry behind MRT enhanced with high-Z materials, including the radiobiological vs. physical differences between MRT peaks and valleys. This study also verified options for the use of DNA damage assays for the bio-dosimetric verification of treatment outcomes for MRT. Accordingly, we have demonstrated a match between the physically prescribed treatment and the biological response of 9LGS cancer, with reduced biological PVDR when MRT is enhanced by radiosensitisers. This match can improve the accuracy of future clinical MRT treatment planning by allowing for a biologically predictable response to a physically prescribed treatment. In particular, the verification of local dose enhancement with radiosensitiser-enhanced MRT can not only improve GBM cancer-killing but also improve MRT tissue-sparing capabilities because a lower dose can be given overall to reduce patient exposure whilst still maintaining treatment efficacy and reliable tumour control.

## Data Availability

Data are available within this paper and in Appendix A. The raw data supporting the conclusions of this article will be made available by the authors on request. All image analysis code functions are available via ImageJ from the National Institutes of Health, United States. All computational functions for dataset analysis and calculations are available in Microsoft Excel from the Microsoft 365 software suite. All were applied to the image analysis of confocal microscopy images in accordance with the methods and protocols listed within this article.

## Appendix A

This Appendix contains all supplementary figures, tables, and supporting information for the main text.

**Table A1:** Dosimetric parameters and beam configurations for SBB and MRT. All technical parameters were used for in vitro studies at the Australian Synchrotron Imaging and Medical Beamline (IMBL). All beam geometry and intrinsic dose rates are measured at the sample position. Conventional broadbeam (CBB) configuration parameters, as described in the Materials and Methods, are shown for comparison of high-dose rate synchrotron broadbeam (SBB) and microbeam radiation therapy (MRT) to the low-dose rate CBB radiotherapy modality.

Mode	Wiggler Field (T)	Filtration (mm)	Mean Energy (keV)	Beam Height (mm); Beam Width (cm); Number of Columns	Intrinsic Dose Rate (Gy/s) in Solid Water	PVDR
CBB	N/A	Be (3.00)	66	N/A	0.0125 at entrance	N/A
		Cu (0.35)				
		Al (1.50)				
SBB	2	Cu (1.41)	71.4	0.500; 0.140; 4	74.1 at 24 mm depth in Solid Water	N/A
		Al (2.82)				
MRT	2	Cu (1.41)	71.4	0.480; 0.385; 4	50.3 (peak), 5.7 (valley) at 24 mm depth	8.9
		Al (2.82)				
